# Staphylococcus lugdunensis does not exert competitive exclusion on human corneocytes

**DOI:** 10.1099/mic.0.001522

**Published:** 2025-01-31

**Authors:** Tianqi Zhang, Ran Luo, Marcus Ehrström, Keira Melican

**Affiliations:** 1 AIMES–Center for the Advancement of Integrated Medical and Engineering Sciences, Karolinska Institutet and KTH Royal Institute of Technology, SE-171 77 Stockholm, Sweden; 2 Department of Neuroscience, Karolinska Institutet, SE-171 77 Stockholm, Sweden; 3 Nordiska Kliniken, Stockholm, Sweden

**Keywords:** MRSA, *S. lugdunensis*, stratum corneum, skin, *S. epidermidis*

## Abstract

Human skin is our primary physical barrier and largest immune organ, and it also hosts a protective microbiota. Staphylococci are prominent members of the skin microbiota, including the ubiquitous coagulase-negative staphylococci (CoNS). The coagulase-positive *Staphylococcus aureus* is found as part of the microbiota, but it poses clinical concern due to its potential pathogenicity and antibiotic resistance. Recently, a CoNS, *Staphylococcus lugdunensis*, has been shown to inhibit *S. aureus* growth via the production of a novel antibiotic, lugdunin. In this study, we use human skin models to understand the spatial relationships between the CoNS *Staphylococcus epidermidis* and *S. lugdunensis* with *S. aureus* during colonization of human skin. We investigated the attachment patterns of the bacteria, both individually and in competition. Surprisingly, we found that attachment did not always correlate with colonization ability. *S. lugdunensis* exhibited significantly reduced attachment to human skin stratum corneum but was an efficient longer-term colonizer. *S. lugdunensis* had a distinct attachment pattern on human corneocytes, with no significant overlap, or competitive exclusion, with the other strains. *S. lugdunensis* is a potential probiotic strain, with a proven ability to suppress *S. aureus*. Before this potential can be realized, however, further research is needed to understand how this strain adheres and interacts with other bacteria in the human skin microenvironment.

## Introduction

The skin is the largest human organ and serves as one of the body’s first-line defences against invasive pathogens [1,4]. A key feature of this barrier is the outermost layer of the epidermis, the stratum corneum, composed of anuclear, terminally differentiated corneocytes and intercellular lipids [4,5]. Human skin is home to a diverse bacterial microbiota believed to actively contribute to skin defence by competitive exclusion of potential pathogens and by educating the local innate immune response [1,3,6]. The staphylococci are one of the most abundant bacterial species of the skin microbiota [6]. The coagulase-negative (CONs) *Staphylococcus epidermidis* is found across multiple skin sites [7], while the clinically important pathogen *Staphylococcus aureus* is referred to as a ‘pathobiont’, a symbiont with significant pathogenic potential [6,8, 9].

To successfully colonize human skin, bacteria must first attach. Previous work with human skin models in our lab highlighted the outer stratum corneum as a primary colonization site for *S. aureus* on human skin [10,11]. Other labs have shown that cell wall-anchored (CWA) protein accumulation-associated protein (Aap) from *S. epidermidis* and the orthologous surface protein G (SasG) from *S. aureus* are important to attachment to healthy human corneocytes [12,14]. As is often the case for staphylococci, there appears to be redundancy in this process. Many *S. aureus* strains, including JE2, appear to possess premature stop codons in the SasG sequence, suggesting that the presence of other binding mechanisms is also likely [14]. CWA clumping factor B (ClfB) and fibronectin-binding protein B (FnBPB) have been implicated in facilitating *S. aureus* colonization of pathologic atopic dermatitis (AD) skin [15,17]. However, as AD skin has very different characteristics, whether similar mechanisms are present in healthy human skin remains to be studied.

Research has investigated differences between the potentially pathogenic *S. aureus* and commensal CoNS often using the prototypical CoNS *S. epidermidis*. More recently, other CoNS have been gaining attention for their role in protecting against pathogens. *S. lugdunensis* has garnered particular consideration following the discovery of its production of a novel antibiotic, lugdunin [18]. *S. lugdunensis* is however far from harmless, it causes skin and soft tissue infections and a particularly aggressive form of infective endocarditis [19,20]. Despite being considered a skin commensal, little is known about the mechanism behind *S. lugdunensis* attachment to human skin. CWA Von Willebrand factor-binding protein (vWbl) and fibrinogen-binding protein (Fbl) have been proposed to facilitate *S. lugdunensis* attachment to the extracellular matrix during endothelial invasion and endovascular infections [21], and the expression of autolysin AtlL promoted *S. lugdunensis* adherence to epithelial and endothelial cells [22]. For skin attachment, however, a specific adhesion has not been described, and *S. lugdunensis* does not encode ClfB and SdrD, nor any orthologue to the *S. epidermidis* Aap [21,23, 24].

In this work, we compared the colonization and attachment of *S. aureus*, *S. epidermidis* and *S. lugdunensis* in a human skin biopsy model and confirmed the outer stratum corneum as the primary binding site for the selected strains. We then used a corneocyte adhesion assay and optimized it for a comparison of competitive exclusion. We investigated how staphylococci differentially attach to the outer stratum corneum and whether any potential space exclusion occurs between the strains. We show that *S. lugdunensis* has a unique binding pattern compared to *S. aureus* and *S. epidermidis* and shows a limited role in competitive exclusion on human corneocytes.

## Methods

### Bacterial strains and culture conditions

The bacterial strains used are listed in Table 1. Strains were grown in Tryptic soy broth (TSB) (Sigma-Aldrich) with indicated antibiotic, at 37 °C with 180 r.p.m. agitation. For log-phase suspension, an overnight culture was diluted 1 : 100 in TSB with appropriate antibiotic and incubated at 37 °C 180 r.p.m. to OD600 ~0.6. The culture was washed twice with PBS using centrifugation (4000 r.p.m., 10 min) and re-suspended PBS to the required OD600.

**Table 1.** Bacterial strains, plasmid

Strains	Description	Selective antibiotics	Source
*S. aureus*	AH3849 - USA300 CA-MRSA, Erm^s^ (LAC*) (AH1263) pHC48 (DsRed)	Chloramphenicol 25 µg ml^−1^	Ibberson et al. (2016) [31]
*S. epidermidis*	*S. epidermidis* 1457 pSRPFS1 (RFP)	Trimethoprim 10 µg ml^−1^	Mack et al. (1992) [32]This work
*S. lugdunensis*	*S. lugdunensis* HKU09-01 pRSA-GFP	Erythromycin 5 µg ml^−1^	Flannagan et al. (2018) [28]
**Plasmids**			
pSRFPS1	Vector for RFP	Trimethoprim	BEI Resources NR-51164

### *Ex vivo* human skin model (biopsy model)

After collection, the skin surface was sterilized with 70% ethanol ×3. Full-thickness *ex vivo* human skin biopsy was collected with an Acuderm Inc. Biopsy Punch 10 mm (Thermo Fisher Scientific). The biopsy was immersed in 70% EtOH for 10 s and washed with PBS, pH 7.4 (Gibco™ Thermo Fisher Scientific). After drying, a hydrocoll (Kontorsgiganten) hydrogel ring was placed on each biopsy to seal the edges. Biopsies were placed in a TC insert (Sarstedt) and each insert into a Corning® Costar® TC-Treated 12-Well Plate (Sigma-Aldrich). Six hundred fifty microlitres of CO_2_ Independent Medium (Thermo Fisher Scientific) supplemented with 1X CTS™ GlutaMAX™-I Supplement (Thermo Fisher Scientific) and 10% foetal bovine serum (Sigma-Aldrich) (culture medium) were added under the biopsies, with the skin surface remaining in air. The biopsies were incubated at 37 °C overnight. Colonization inoculum was prepared by diluting a log-phase bacterial suspension with PBS to an OD600 1.95 (~ 6.5×10^8^ c.f.u. ml^−1^). Two microlitres of the colonization inoculum were evenly distributed across the biopsy surface. The biopsy was incubated at 37 °C for 48 h with media changes every 24 h. After incubation, the hydrogel ring was removed, and the biopsy was collected for subsequent analysis.

### Tape-stripped human skin stratum corneum model

The surface of the skin biopsy was taped once with Office Tape 31-3278 (Clas Ohlson) to remove debris. To isolate stratum corneum, dermatology tape d-Squame Standard 1up Label (Clinical and Derm) was applied to the skin surface with consistent pressure for 5 s. The tape was gently removed and placed cell-side up in a Corning® Costar® TC-Treated 6-Well Plate (6-well plate) (Sigma-Aldrich).

### Bacterial attachment assays

For attachment assays, the inoculum was diluted from log-phase suspension with PBS to OD600 0.15 (~ 5×10^7^ c.f.u. ml^−1^). For the competition assay, attachment inoculum was prepared to an OD600 1.5 (~ 5×10^8^ c.f.u. ml^−1^) for *S. lugdunensis* and OD 0.15 for * S. epidermidis* or *S. aureus*. Three hundred microlitres of inoculum were placed in each well of a 12-well plate. The biopsy was placed onto the drop with the outer skin surface facing downward. For the tape model attachment assay, 300 µl of inoculum was added to the centre of each tape. After 45 min at 37 °C, unbound bacteria were removed by washing extensively with PBS. For the biopsy model, the skin surface was washed with 10 ml PBS, and the bottom was washed with 2 ml PBS. For the tape model, the cell side was washed with 10 ml PBS, and the backside was washed with 2 ml PBS.

### c.f.u. quantification

Biopsies or tapes were homogenized using Lysing Matrix D (MP Biomedicals) tube with 1 ml PBS. Tubes were homogenized at 6.5 m/s for 20 s ×3, with 5-min interval on ice. The homogenate was serial diluted and plated on a Tryptic soy agar (TSA) (Sigma-Aldrich) containing selective antibiotics as outlined in Table 1 and incubated at 37 °C overnight. To facilitate analysis, a c.f.u.=0 is transformed to 1, which represents the limit of detection of this model. To stabilize variance, the c.f.u. numbers were transformed into logarithmic base 10 values (log-transformation) and plotted using Prism 10 software (GraphPad). Statistical comparisons between the log-transformed results were conducted with Prism 10 using unpaired *t*-tests for comparisons between two groups and ordinary one-way ANOVA with Tukey correction for comparisons involving more than two groups.

### Imaging analysis

Biopsies were placed in an Epredia™ Disposable Embedding Mold (Fisher Scientific) and embedded in CellPath OCT Embedding Matrix (Fisher Scientific). The embedded biopsy was flash-frozen and stored at −80 °C. Prior to staining, the OCT-embedded biopsy was sectioned 20 µm, mounted on VWR® SuperFrost® Plus slides (VWR) and fixed with 100% methanol (Sigma-Aldrich) on ice for 10 min. Immunofluorescence staining utilizing the following antibodies: NucBlue™ Fixed Cell ReadyProbes™ Reagent (DAPI) (R37606, Thermo Fisher Scientific) and Alexa Fluor™ 647 Phalloidin (A22287, Thermo Fisher Scientific). Primary antibodies were incubated at 4 °C overnight, while secondary antibodies, Alexa Fluor™ 700 (A-21038, Thermo Fisher Scientific), were incubated at room temperature for 1 h. Following staining, the sample was mounted against 50×24 mm VWR® Micro Cover Glasses (50×24 cover glass) (VWR) using Dako Fluorescence Mounting Medium (Agilent) and stored overnight at 4 °C before imaging.

### Processing and staining of the tape

For visualization of bacterial attachment to the tape model, each tape was directly mounted onto a 50×24 mm cover glass and sealed against a microscopy slide (VWR). Following fixation, the cell side of the tape was stained using the stains listed above. Once stained, the tape was fitted and mounted in µ-Dish 35 mm, high (Ibidi) using Dako Fluorescence Mounting Medium. All mounted samples were imaged immediately.

### Microscopy

Samples were imaged using an LSM 700 Laser Scanning Microscope (Zeiss) with a 20× air objective. Post-acquisition, images underwent minor adjustments to brightness and contrast, uniformly applied to the entire image and digitally enlarged using Fiji software (ImageJ, National Institutes of Health, USA).

## Results

### Different *Staphylococcus* strains colonize and adhere differently to human skin

**Fig. 1.**
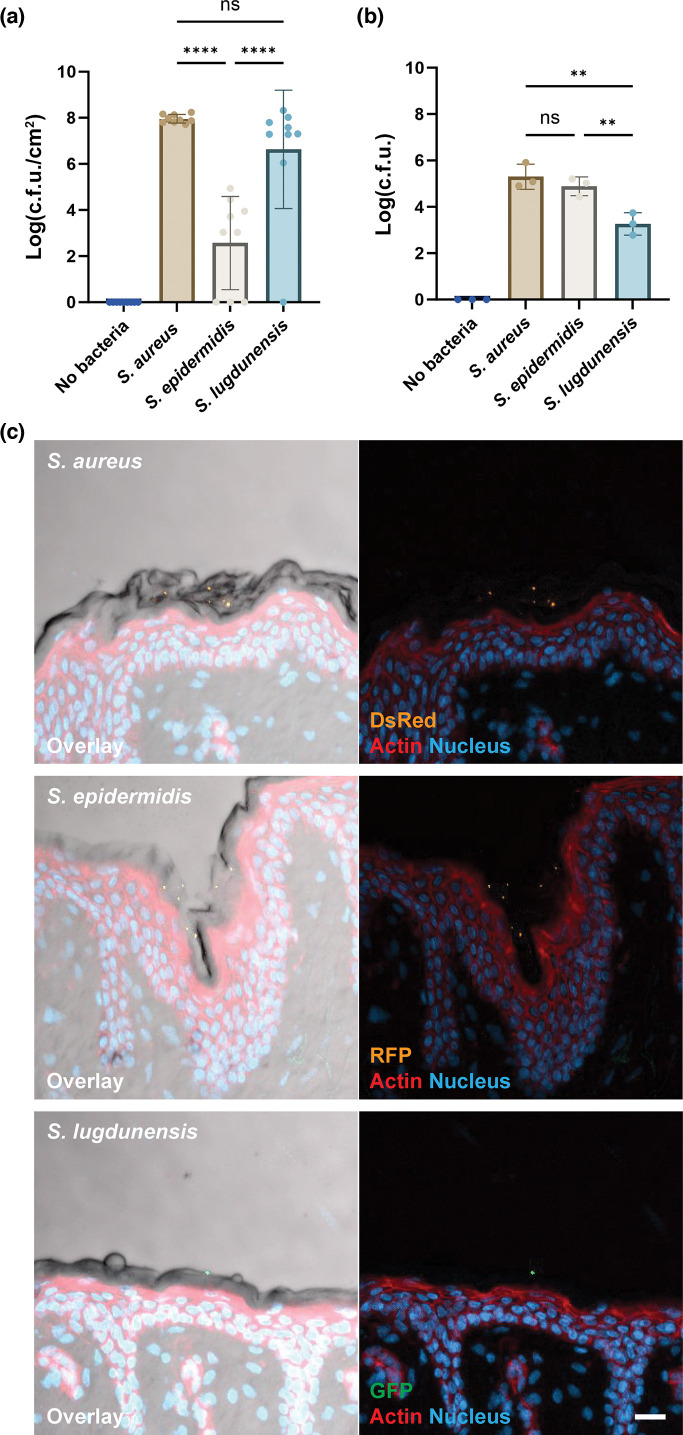
Staphylococci colonization and attachment to human skin biopsy. (**a**) Colonization of human skin at 37 °C for 48 h. Mean log transformed c.f.u. per cm^2^ with sd. *n*=9 from three individual skin donors. Statistical significance was determined by one-way ANOVA. Statistical significance is denoted by ns (not significant) or ****P≤0.0001. (**b–c**) Attachment of the selected strains at 37 °C for 45 min. (**b**) Mean log-transformed total c.f.u. is shown. Combination of *n*=3 from three individual donors. Statistical significance was determined by one-way ANOVA. Statistical significance is denoted by ns (not significant) or **P≤0.01. (**c**) Fluorescence imaging of the location of bacterial attachment on human skin. DsRed-expressing *S. aureus* (AH3849), RFP-expressing *S. epidermidis* (1457-pSRFPS1) and GFP-expressing *S. lugdunensis* (*S. lugdunensis*-GFP) are shown. Overlay image is a combination of brightfield, DsRed channel (DsRed or RFP) in orange, GFP channel (GFP) in green, Alexa 647 channel (actin) in red and DAPI channel (nucleus) in blue. Representative images of *n*=3. Scale bar=20 µm.

As an extension to our previous work on the human skin colonization patterns of *S. epidermidis* vs. *S. aureus* [11], we included here *S. lugdunensis*. We inoculated *ex vivo* human skin biopsies with 1.3×10^6^ c.f.u. of *S. aureus*, *S. epidermidis* or *S. lugdunensis* and incubated at 37 °C for 48 h. After 48 h, *S. aureus* and *S. lugdunensis* grew on the skin, colonizing the surface, with *S. aureus* showing a mean log_10_ c.f.u. cm^−2^ of 8.0 and *S. lugdunensis* 6.6 (Fig. 1a). *S. epidermidis* demonstrated significant donor-to-donor variation in its colonization, something we have noted in previous work [11], with a significantly lower mean log_10_ c.f.u. cm^−2^ of 2.6 (Fig. 1a). These data showed a variance between the colonization properties of the different bacterial strains on the skin biopsy model at 48 h, suggesting *S. lugdunensis* colonized more like *S. aureus* than *S. epidermidis*. The first step in colonization is bacterial attachment. We, therefore, sought to quantify bacterial attachment to the human skin biopsy. Using methodology based on previous assays [12,14], we adhered the bacteria to the skin surface for 45 min before extensive washing. Interestingly, the results of the adhesion assay differed from colonization. *S. lugdunensis* demonstrated the lowest level of adhesion with a mean of 3.3 log_10_ c.f.u., significantly lower than both *S. aureus* (5.3) and *S. epidermidis* (4.9) (Fig. 1b). Individual donor variation was again evident. *S. lugdunensis* showed a 100-fold reduction in adhesion compared to *S. aureus* and a 40-fold decrease compared to *S. epidermidis* (Fig. 1b). Microscopy analysis showed that *S. aureus*, *S. epidermidis* and *S. lugdunensis* all adhered primarily to the stratum corneum (Fig. 1c), consistent with our previous work [10,11]. These results suggest an apparent discrepancy in staphylococcal adhesion vs. colonization on human skin. *S. aureus* showed a strong ability to attach and colonize the human skin biopsy models, while *S. epidermidis* was able to attach but was slower to colonize. *S. lugdunensis* had a lower ability to attach but colonized the skin to a comparable level as *S. aureus*.

### Staphylococcal attachment to isolated corneocytes

**Fig. 2.**
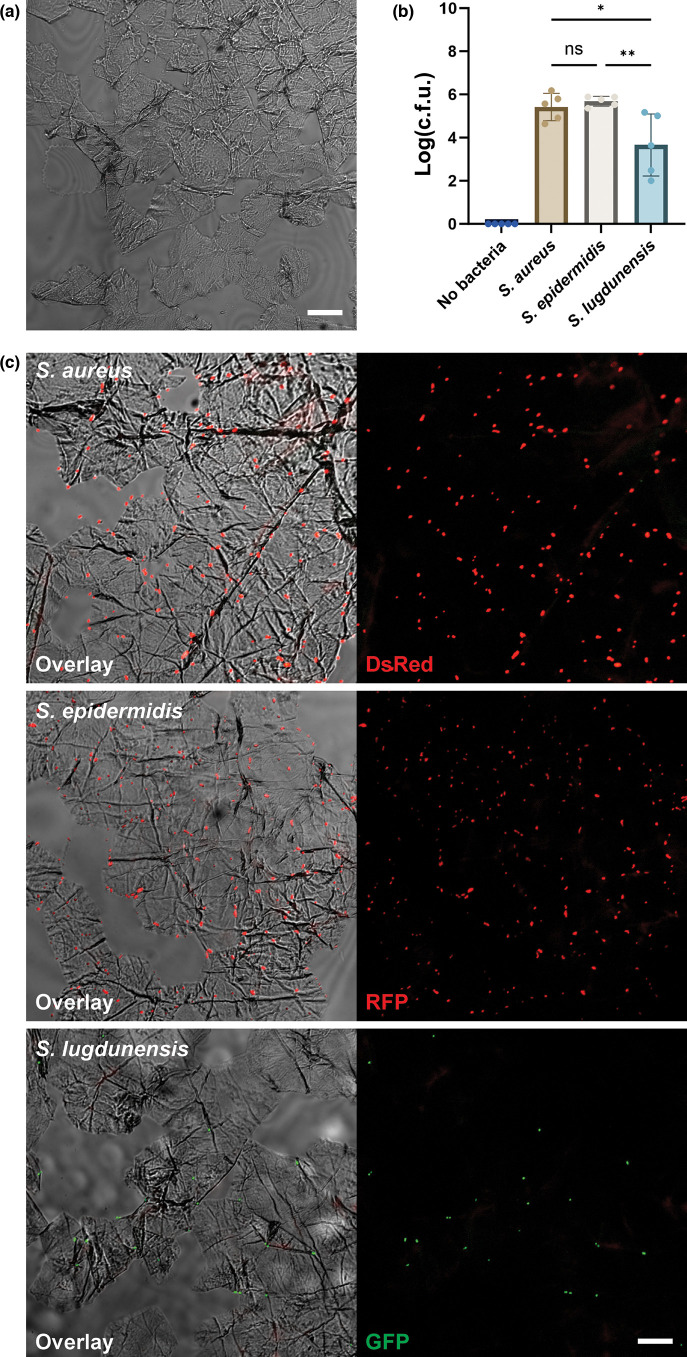
Staphylococci attachment to isolated corneocytes. (**a**) Brightfield imaging of uninfected tape-stripped stratum corneum. Representative of *n*=3 from three donors. Scale bar=20 µm. (**b–c**) Attachment of the selected strains at 37 °C for 45 min. (**b**) Mean log-transformed total c.f.u. Combination of *n*=5 from five donors. Statistical significance was determined by one-way ANOVA. Statistical significance is denoted by ns (not significant), *P≤0.05 or **P≤0.01. (**c**) Fluorescence imaging of the attachment. DsRed-expressing S. aureus (AH3849), RFP-expressing S. epidermidis (1457-pSRFPS1) and GFP-expressing S. lugdunensis (S. lugdunensis-GFP) were shown. The overlay image was generated in combination with the brightfield channel, DsRed channel (DsRed or RFP) in red and GFP channel (GFP) in green. Representative images of *n*=3 from three donors. Scale bar=20 µm.

To assess the direct role of the stratum corneum in bacterial adhesion, we focused on bacterial attachment to primary corneocytes. We adopted a previously used approach, using tape-stripped human skin to isolate the stratum corneum [25,26] (Fig. 2a). We repeated the attachment assay using freshly isolated tape-stripped corneocytes. Forty-five minutes after exposure of corneocytes to bacteria, the cells were washed with PBS to remove any non-adherent bacteria. Bacterial adhesion to isolated corneocytes reflected what we had seen in the full-thickness biopsy (Fig. 2b). *S. aureus* (5.4 mean log_10_ c.f.u.) and *S. epidermidis* (5.7) adhered to a similar degree, while *S. lugdunensis* again demonstrated significantly lower adhesion (3.7 log_10_ c.f.u.) (Fig. 2b). To visualize the binding patterns of the staphylococci on corneocytes, we performed fluorescence imaging. Microscopy of uninfected tape-stripped cells confirmed that the majority of cells isolated by our model are anuclear skin corneocytes (Fig. 2a). Microscopy revealed a dense presence of *S. aureus* and *S. epidermidis* on the corneocytes even after extensive washing (Fig. 2c). In contrast, *S. lugdunensis* showed considerably less binding. Interestingly, most of the staphylococci found in this model were adhering at the edges of the corneocytes, at the cell–cell borders (Fig. 2c). Together, these data showed similarity in bacteria attachment patterns to isolated corneocytes compared to the full-thickness biopsy. The binding location of most bacteria at the cell–cell junctions of the corneocytes highlighted potential binding preference towards binding targets present at the edges of the corneocytes.

### Limited competitive exclusion on human corneocytes

**Fig. 3.**
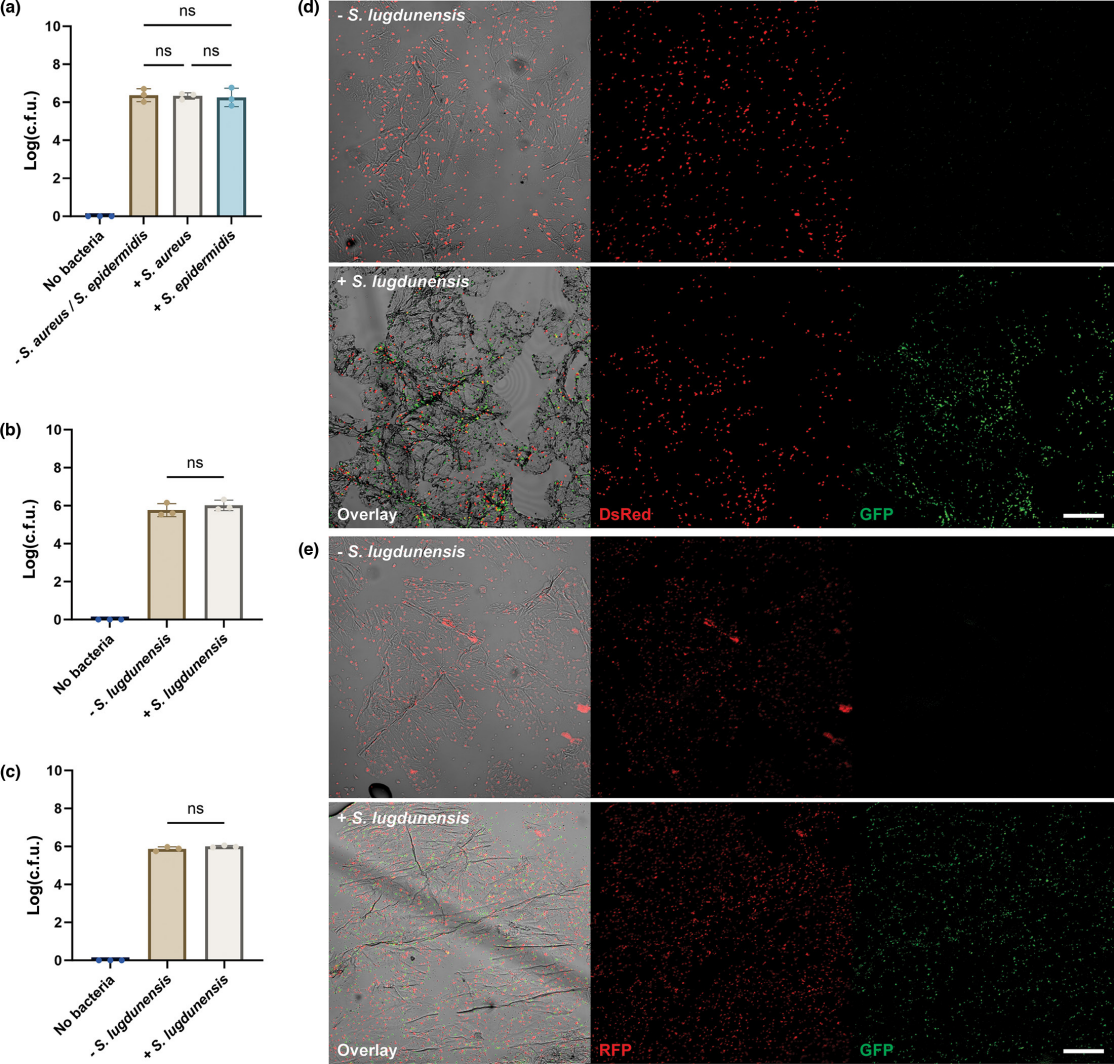
Staphylococci competition on isolated corneocytes. (**a**) *S. lugdunensis* attachment was not affected by the presence of *S. aureus* or *S. epidermidis*. (**b, c**) The presence of *S. lugdunensis* did not affect the attachment of (**b**) *S. aureus* or (**c**) *S. epidermidis*. Mean log-transformed total c.f.u. is shown. *n*=3 from three donors. Statistical significance was determined by one-way ANOVA. Statistical significance denoted by ns (not significant), *P≤0.05 or **P≤0.01. (**d, e**) Fluorescence imaging the attachment of (**d**) *S. aureus* or (**e**) *S. epidermidis* without (top) and with (bottom) the presence of abundant *S. lugdunensis*. DsRed-expressing *S. aureus* (AH3849), RFP-expressing *S. epidermidis* (1457-pSRFPS1) and GFP-expressing *S. lugdunensis* (*S. lugdunensis*-GFP) are shown Overlay image is a combination of brightfield, DsRed channel (DsRed or RFP) in red and GFP channel (GFP) in green. Representative images of *n*=3 from three donors. Scale bar=50 µm.

Little is known regarding the binding mechanism of *S. lugdunensis* to human skin. Previous studies have shown that CWA proteins such as accumulation-associated protein (Aap) from *S. epidermidis* and its orthologous surface protein G (SasG) from *S. aureus* are critical to staphylococci attachment to corneocytes [25]. However, no orthologue to Aap or SasG has yet been found in *S. lugdunensis* [19,23]. We, therefore, sought to understand if there may be a functional orthologue in *S. lugdunensis* by designing a competitive exclusion assay. We co-attached *S. aureus* or *S. epidermidis* together with a tenfold increased concentration of *S. lugdunensis*. To start, we increased the inoculum of *S. lugdunensis* to OD600 1.5 (~ 5×10^8^ c.f.u. ml^−1^) (Fig. 3a). The presence of neither *S. aureus* nor *S. epidermidis* in competition altered the adhesion numbers of *S. lugdunensis* (Fig. 3a). Comparably, the large *S. lugdunensis* volume did not alter the adhesion capability of *S. aureus* with mean log_10_ c.f.u. of 5.9 when attaching individually (Fig. 2b) and 6.0 when in competition with *S. lugdunensis* (Fig. 3b). No statistical difference was seen between the two conditions, suggesting limited competitive exclusion. Similar results were seen for competition between *S. epidermidis* and *S. lugdunensis. S. epidermidis* showed a mean log_10_ c.f.u. of 5.8 while attaching alone and 6.0 while attaching in competition with *S. lugdunensis*. No statistical difference was found between the two conditions (Fig. 3c). Microscopy of the competition assay confirmed the quantification (Fig. 3d, e). Similar amounts and patterns of * S. aureus* binding could be seen regardless of *S. lugdunensis* presence (Fig. 3d). This was also the case for *S. epidermidis* (Fig. 3d, e). Taken together, no competitive exclusion was found between *S. lugdunensis* and *S. aureus* or *S. epidermidis* in this model. This indicates that *S. lugdunensis* has a unique adhesion pattern on human corneocytes.

## Conclusions

Adhesion is often the first step of host–bacterial interaction. The understanding of how different bacteria adhere, bind and colonize different tissues is an ongoing research question and a promising target for novel antimicrobial therapies. The protective properties of the human microbiota are believed to involve competitive exclusion, where the resident microbiota outcompete potential pathogens for space and nutrients. In this work, we focused on the potential skin pathogen *S. aureus* and the interaction with both human skin and CoNS. It has become apparent in recent years that there is great variation among the CoNS, so we included the prototypical skin commensal *S. epidermidis* as well as the emerging potential probiotic strain *S. lugdunensis*. The colonization and adhesion patterns of *S. aureus* and *S. epidermidis* were similar to previous results from our own work as well as by others [10,13, 26, 27]. Particularly, we see significant donor-to-donor variation in *S. epidermidis*, which we believe relates to the background of the individual donors and their prior exposure to skin microbiota. While the use of human skin does introduce this variance, we believe it is more representative of the real-world situation. *S. lugdunensis* gave interesting results, showing that while it appears to colonize over 48 h at a similar extent to * S. aureus*, its adhesion at 45 min was significantly less. *S. lugdunensis* is an intriguing CoNS with very particular metabolic requirements, the best described being its iron-acquisition systems [28,29]. It is the only CoNS to express an iron-regulated surface determinant system (Isd) and can use this high-affinity uptake system in low-haem environments such as the skin. Distinctively, the *S. lugdunensis* IsdB has a reduced affinity to murine haemoglobin compared to humans, indicating a human specificity of this bacteria [19,28]. Our data appear to support this specificity, showing an unexpectedly good colonization rates on human skin for *S. lugdunensis*, showing a colonization pattern more similar to *S. aureus* than *S. epidermidis*. Emerging data from other labs as well as our own preliminary data suggest a significant interplay between *S. lugdunensis* and *S. aureus*. This included how *S. lugdunensis* produces the antibiotic lugdunin [18] as well as data showing that *S. lugdunensis* can hijack iron acquisition and nutritional immunity of *S. aureus* to facilitate growth [19,30]. Our data support this exciting line of research by demonstrating for the first time that the adhesion patterns of * S. lugdunensis* are significantly different from both *S. aureus* and *S. epidermidis* on human skin. This would suggest that its inhibitory properties are not related to competitive exclusion. Our data show that *S. lugdunensis* has a unique binding pattern compared to the other strains that correspond to other data, showing that *S. lugdunensis* does not possess many of the known CWA proteins present in the other staphylococci such as ClfB, SdrD and Aap [19]. Further research is now needed to define this target. Lastly, our data provide information regarding the spatial localization of the different staphylococcal strains on human skin. This is important for the ongoing work into contact-dependent inhibition, quorum repression and iron acquisition as potential treatment targets. In conclusion, this work provides new knowledge on the binding targets and patterns of different staphylococcal strains on human skin. It opens several new avenues for research into the interactions between staphylococcal strains and how we can take advantage of the human skin microbiota as a means to prevent infection.

## Abbreviations

AD: atopic dermatitis
CoNS: coagulase-negative staphylococci
CWA: cell wall-anchored
TSB: tryptic soy broth
